# Describing knowledge encounters in healthcare: a mixed studies systematic review and development of a classification

**DOI:** 10.1186/s13012-017-0564-1

**Published:** 2017-03-14

**Authors:** Dominic Hurst, Sharon Mickan

**Affiliations:** 1 Barts and The London School of Medicine and Dentistry, Queen Mary University of London, London, UK; 2 Griffith University, Gold Coast, Queensland Australia; 3 Gold Coast Health, Gold Coast, Queensland Australia; 4 Nuffield Department of Primary Care Health Sciences, University of Oxford, Oxford, UK

**Keywords:** Knowledge encounters, Information sources, Healthcare workers, Faceted classification, Mixed studies, Systematic review, Evidence-based practice

## Abstract

**Background:**

Implementation science seeks to promote the uptake of research and other evidence-based findings into practice, but for healthcare professionals, this is complex as practice draws on, in addition to scientific principles, rules of thumb and a store of practical wisdom acquired from a range of informational and experiential sources. The aims of this review were to identify sources of information and professional experiences encountered by healthcare workers and from this to build a classification system, for use in future observational studies, that describes influences on how healthcare professionals acquire and use information in their clinical practice.

**Methods:**

This was a mixed studies systematic review of observational studies. DATA SOURCES: OVID MEDLINE and Embase and Google Scholar were searched using terms around information, knowledge or evidence and sharing, searching and utilisation combined with terms relating to healthcare groups. ELIGIBILITY: Studies were eligible if one of the intentions was to identify information or experiential encounters by healthcare workers. DATA EXTRACTION: Data was extracted by one author after piloting with another. STUDY APPRAISAL: Studies were assessed using the Mixed Methods Appraisal Tool (MMAT). PRIMARY OUTCOME: The primary outcome extracted was the information source or professional experience encounter. ANALYSIS: Similar encounters were grouped together as single constructs. Our synthesis involved a mixed approach using the top-down logic of the Bliss Bibliographic Classification System (BC2) to generate classification categories and a bottom-up approach to develop descriptive codes (or “facets”) for each category, from the data. The generic terms of BC2 were customised by an iterative process of thematic content analysis. Facets were developed by using available theory and keeping in mind the pragmatic end use of the classification.

**Results:**

Eighty studies were included from which 178 discreet knowledge encounters were extracted. Six classification categories were developed: what information or experience was encountered; how was the information or experience encountered; what was the mode of encounter; from whom did the information originate or with whom was the experience; how many participants were there; and where did the encounter take place. For each of these categories, relevant descriptive facets were identified.

**Conclusions:**

We have sought to identify and classify all knowledge encounters, and we have developed a faceted description of key categories which will support richer descriptions and interrogations of knowledge encounters in healthcare research.

**Electronic supplementary material:**

The online version of this article (doi:10.1186/s13012-017-0564-1) contains supplementary material, which is available to authorized users.

## Background

### Implementation science and clinical practice

Well-evidenced interventions intended to improve patient well-being may not be used optimally across healthcare professions [1–8] with many barriers identified to the uptake of best evidence [5, 6, 9–12] and interventions designed to overcome these [13–15]. Implementation science seeks to promote the uptake of research and other evidence-based findings into practice [16], but for healthcare professionals, this is complex as practice draws on, in addition to scientific principles, rules of thumb and a store of practical wisdom [17] acquired from a range of informational and experiential sources. Gabbay and Le May’s ethnographic study of how primary care clinicians build *mindlines*—“collectively reinforced, internalised, tacit guidelines”—found it was rare that medical practitioners searched for guidelines [18, 19]. Instead, clinicians drew on multiple sources of information and experiences as they cared for patients. Much of the knowledge they acquired was tacit, i.e. it could not be put into words [20], as, for example, when someone tries to convey to another exactly how *not* to fall off a bike when riding. The knowledge is embodied. We know *how* to cycle and yet cannot fully explain it. So, rather than conceiving of a single knowable reality, mindlines are based on a more “fluid, embodied and intersubjective view of knowledge” that accommodates context and multiple realities [21]. In this way, the knowledge of the practitioner is in the moment and in their practice (or “knowledge-in-practice”) in a particular context of space and time (“knowledge-in-practice-in-context”) [18]. Implementation science, with its aspiration to change practice, could benefit from a richer understanding of how the complex, personal and context-laden phenomenon of knowledge-in-practice develops over time.

### Existing reviews of information sources used by clinicians

A number of reviews have sought to collate the sources of information clinicians use and the ways in which they seek them [22–28]. These include the information sources used by rural health professionals [27], nurses [25], physicians [22, 23, 28] and dentists in developed countries [24]. The reviews found that colleagues were often ranked as primary information sources and that learning informally was widespread. However, none of them attempted to synthesise the research from across healthcare groups, and none attempted to classify the context and other attributes of healthcare professionals’ interactions with information or experiences. Thus, implementation research is not yet able to explore what characteristics are associated with the information and experiential encounters that matter to healthcare professionals.

### Defining “knowledge encounters”

Our prior reading suggested that in order to describe the breadth of occasions when clinicians come across phenomena that have the potential to change their knowledge, the more familiar terms in the literature around knowledge transfer, translation, exchange and sharing would not be sufficient. They could not account for the unplanned way in which the mindlines work suggested healthcare professionals developed their knowledge-in-practice. We found “encounter” to be a broad enough term to help describe these occasions as it may be expected or unexpected, brief or protracted, experiential and involve a degree of “dealing with” something or someone [29–31].

Many definitions of knowledge exist but we adhered to an interpretivist perspective of knowledge as discussed above in the context of mindlines and knowledge-in-practice-in-context. Working from Stenmark [32], we conceive of information as a means (e.g. written, oral, performative) of attempting to articulate an individual’s knowledge, but it lacks the personal, tacit, understanding that the individual has of the particular phenomenon they describe. Experiential knowledge, on the other hand, is that knowledge gained through observations in routine practice, for example, of what does and does not “work” [33] and is often followed by a period of sense-making [34]. Information, from Stenmark’s perspective, may *alter* another individual’s prior knowledge but is not in itself knowledge.

We therefore defined a knowledge encounter as a circumstance in which an individual interacts with information or an experience that has the potential to influence their knowledge-in-practice. Knowledge here, therefore, is the potential for knowledge to change in *response* to the information or experience, rather than being an encounter *with* knowledge.

### Aims

The aims of this review were the following:

- To identify the sources of information and professional experiences (referred throughout this article simply as “experiences”) encountered by healthcare workers that could influence their knowledge-in-practice and the contexts within which they are encountered
- To build a classification system, for use in future observational studies, that describes influences on how healthcare professionals acquire and use information in their clinical practice

## Methods

### Identification of studies

A systematic mixed studies review was conducted to identify the information sources and experiences that health professionals reportedly encounter. “Mixed studies reviews” include quantitative, qualitative and mixed methods studies to provide a more holistic view of a given problem [35, 36].

### Study designs

Observational studies in English or German that sought to identify sources of information and experiences, and the ways in which these were encountered, were eligible. Studies that only explored a narrowly restricted source, e.g. internet, were excluded. Experimental study designs were excluded because we were interested in gathering data from existing practice rather than a modified one.

### Participants

All healthcare professionals responsible for patient (including animal) care were eligible. Studies involving a majority of undergraduate students were not eligible. We defined as eligible healthcare professionals any clinical professional that fell within the MEDLINE MeSH term “Health Occupations”. This includes: allied health occupations (e.g. occupational therapy), chiropractors, dentists, medical doctors, nurses, optometrists, podiatrists and veterinary practitioners. We included all of these, including veterinary practitioners, because we felt that whilst there are differences in the contexts within which information or experiences are encountered, the underlying process of professional learning and development are sufficiently similar across professional settings.

### Outcomes

Any information source or experience related to patient care (this includes the care of animals in the case of veterinarians) was eligible.

### Electronic databases and search engines

The following databases and search engines were searched: OVID MEDLINE, OVID Embase, and Google Scholar. Embase was searched from the first records in 1974 to June 6, 2014. Medline was searched from 1946 to week 4 of May 2014 using terms relating to information, knowledge or evidence and sharing, searching and utilisation combined with terms relating to the various healthcare groups. [For search strategy, see Additional file 1]. The final search was conducted on February 6, 2014. Google Scholar was searched using the combinations of “sources of knowledge” and healthcare groups. The searches were run again on July 29, 2016, to identify studies that included any information or knowledge sources not identified in the studies from the original search.

### Reference lists and citation searches

We carried out backward citation searches for all included studies in Google Scholar and forward and backward citation searches in Web of Science for the more comprehensive studies [19, 33, 37] and 10 systematic reviews [22, 23, 25–28, 38–41]. Bibliographies and reference lists were checked for potentially relevant studies’ books on implementation science and evidence-based healthcare in the authors’ personal collections.

### Combining search results and identifying eligible studies

Potentially eligible studies were identified from the databases and Google Scholar searches, exported to EndNote X6 and duplicates removed.

Titles and abstracts of all studies were screened for potential inclusion in the review by one author (DH). Where a study was potentially eligible, the full text was retrieved and the final decision on eligibility made by DH.

### Data extraction

A purpose designed data extraction form was used to extract data from eligible studies. This was piloted by DH and SM. The final data extraction was completed by DH. From eligible studies, the following were extracted: Author; contact details; citation; year; study design; whether study was reliant on recall, real-time data collection or both; instrument/tool/method used and, in the case of quantitative instruments such as questionnaires, the status regarding validation and reliability; study period if longitudinal; participant description (number of participants, healthcare profession); setting—primary, secondary or tertiary; sources of knowledge; themes or categories used/developed by the authors; types of knowledge considered, i.e. tacit, explicit or both; and references of potentially relevant studies or reviews.

The sources of information or experiences were collated in a spreadsheet by DH. Where descriptions of sources were different but shared the same meaning, these were grouped together by DH as a single construct.

The quality of the included studies was evaluated using the Mixed Methods Appraisal Tool (MMAT) [36, 42].

### Analysis

Our preliminary reading of the literature suggested that information sources and experiences, and the way they are encountered, are multifaceted and would benefit from a classification system that could accommodate this. Kwasnik states that “classification is the meaningful clustering of experience” and argued that the way we classify knowledge can assist in discovery of new knowledge [43]. Our intention is that the classification system we develop would help to organise the experience healthcare workers have of encountering new information and experiences and, therefore, assist with the discovery of new methods for implementation science.

We chose to adopt the facet classification approach to describe the important contexts within which information and experiences are encountered because facet classifications recognise that there is more than one way to view the world and that facets themselves are flexible to accommodate new phenomena over time [43]. This approach has been used to classify information on the World Wide Web [44] and in information-seeking tasks [45]. An example of a facet classification is the way we classify wines using the categories of country (France, Germany, Australia, Chile, etc.), grape variety (chardonnay, cabernet, merlot, etc.), alcohol content (0% through 15%) and colour (white, red, rosé).

To describe and organise the experiential and information sources, we adapted the Bliss Bibliographic Classification System, known as BC2. This system classifies, for any piece of knowledge, *what* is being done, *what* are its parts or properties, *how* is this achieved, *by what means* and by *whom*, *where*, and *when*? [46] and was developed from the work of Ranganathan who identified repeating elements of all subjects: personality, matter, energy, space and time [47].

After extracting the data, we developed a classification of knowledge encounters together with a description of facets that described key elements of information and experiential knowledge sources that healthcare workers encountered. We used a constant comparative method with extensive discussion between the authors as the classification was refined.

Our synthesis involved a mixed approach using the top-down logic of the BC2 classification to generate thematic categories and a bottom-up approach to develop facets (or codes) for each category from the data. That is, we altered, added or removed the specific terms used in the BC2 classification, for example from “What is being done” to “What information or experience is encountered”. We then read and re-read the extracted sources, using the constant comparison technique to develop descriptive codes. As an example, when an article reported “colleagues via internet”, we recognised that “someone” was involved in the encounter. Similarly, someone was involved in an article that reported “mentor” and another that included “patients’ experiences of illness”. Thus, we created a category to describe someone from whom a healthcare worker might have obtained information in their knowledge encounter. We called that category “from whom did the information originate or with whom was the experience”, to fit the BC2 logic. Then we re-read our data to generate facets for each category. The facets were higher level descriptive codes of the people involved in the encounter. For example, from the following raw data, we created the facet “practitioner”: “peers”, “observation of others’ practice”, “formalised supervision”, “systematic self-evaluation”, “specialist” and “email contact with specialist”. In a similar way, the facets of “Non-practitioner (colleague)”, “Patient”, “Researcher”, “Educator”, “Regulator”, “Employer” and “Salesperson” were generated.

## Results

### Study characteristics

**Fig. 1:**
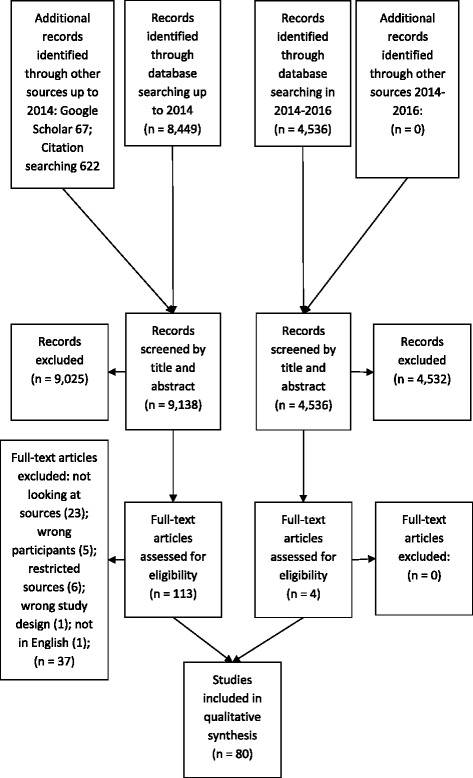
PRISMA flow chart of study selection process

Nine thousand one hundred thirty-eight potentially eligible reports were screened by title and abstract. Of these, 113 were potentially eligible, and therefore, the full text was retrieved. 37 of these were not eligible. 76 studies were eligible for inclusion in the review from the 2014 search [19, 33, 37, 48–120]. The additional search in 2016 identified a further 4 eligible studies [121–124]. Their data are included in the analysis for completeness but they did not identify any additional descriptions of knowledge encounters and did not, therefore, affect the classification (see Fig. 1).

Four studies used a mixed methods design (interviews and surveys), 22 used qualitative methods (ethnography, case study, vignettes, interviews, focus groups or qualitative questionnaire) and 54 used quantitative methods (survey, observation, critical incident technique or interviews). Of the 80 studies, only 24 allowed for the inclusion of knowledge encounters that were experiential or tacit.

Seventy-one of the studies included a single group of healthcare professionals, and 9 involved two or more healthcare groups. Across the studies as a whole, the quality was evaluated by MMAT as moderate or low quality. The study characteristics are summarised in Table 1. [Please see Additional file 2 for details of individual studies].

**Table 1:** Characteristics of included studies

Study design	Mixed methods	4
	Qualitative	22
	Quantitative	54
Cross-sectional or longitudinal	Cross-sectional	76
	Longitudinal	3
	Unclear	1
Recall or real-time	Recall	72
	Real-time	1
	Recall and real-time	7
Setting	Hospital only	22
	Primary care only	28
	Hospital and primary care	15
	Any setting or unspecified	15
Country	Australia	6
	Brazil	1
	Canada	9
	Denmark	1
	France	1
	Ghana	1
	Germany	2
	Iran	4
	Ireland	2
	Italy	1
	Jordan	1
	Mongolia	1
	New Zealand	1
	Norway	2
	Philippines	1
	South Korea	1
	Spain	1
	Sweden	4
	Taiwan	1
	Tanzania	1
	Turkey	2
	UK	11
	USA	25
Healthcare professional groups included across the studies (greater than 80 as some studies included more than one group)	Dentists	8
	Doctors	42
	Radiologists	1
	Nurses	31
	Hygienists	1
	Midwives	1
	Pharmacists	1
	Phlebotomists	1
	Podiatrists	1
	Psychiatrists	1
	Rehabilitation and physical therapists	2
	Speech and language pathologists	1
	Vets	1
Knowledge sources included	Explicit (codified) only	56
	Explicit and tacit or experiential	24
MMAT quality score (maximum ****, minimum *)	****	6
	***	20
	**	37
	*	17

### Development of a classification of knowledge encounters

One hundred seventy-eight individual descriptions of encounters with information sources or experiences were identified across the 80 studies (see Additional file 3). Some of these were described simply as nouns, e.g. *journal*, but there is an implicit requirement for a verb, i.e. to *read a journal*. On other occasions, the verb was recorded, e.g. *informal conversation with colleagues*. Thus, what authors described as “sources” were often “encounters with sources” and might be intentional or unintentional.

**Fig. 2:**
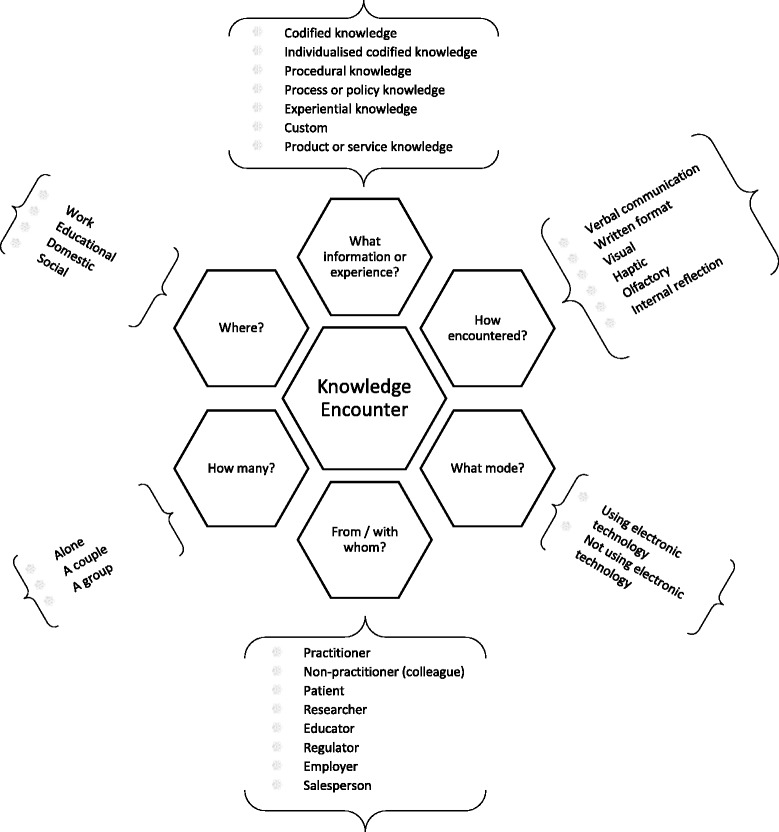
Six categories of knowledge encounters with descriptive facets

We modified the BC2 classification to add categories where differentiation was required and remove redundancies. In summary, the classification we developed included the following six key questions about the knowledge encounter (short versions used in Fig. 2 are in brackets):

- What information or experience was encountered (“What information or experience?”)?
- How was the information or experience encountered (“How?”)?
- What was the mode of encounter (What mode?)?
- From whom did the information originate or with whom was the experience (“From / with whom?”)?
- How many participants were there (“How many?”)?
- Where did the encounter take place (“Where”)?

We removed the BC2 categories “what are its [the knowledge’s] parts or properties” and “when”.

### Facets of categories

Whilst multiple facets were recognised in the literature, we have organised them to describe the important components of the six key classification questions just identified.

#### What information or experience was encountered?

This category reflected the knowledge that the information attempted to articulate, or experiential knowledge. We reviewed existing models of knowledge (see the “Discussion” section) to help construct the facets of this category (Table 2).

**Table 2:** A classification of knowledge encounters: knowledge facets, descriptions and examples

Knowledge facet	Description	Examples from included studies
Codified knowledge	Research, theoretical or practice-based knowledge subject to quality control by editors, peer review and debate	Journal, guidelines, textbook
Individualised codified knowledge	Codified knowledge presented in some manner by individuals in person	Informal conversation with colleagues, seminars, grand rounds
Procedural knowledge	The individual actions required to carry out a given activity	Observation of other’s practice
Process or policy knowledge	Local or national processes and policies	Local care delivery, national health policy
Experiential knowledge	That obtained through personal experience	What has worked/not worked before, personal clinical experience, patient’s experiences of illness
Custom	The implicit norms of a given healthcare setting or professional group	The way it has always been done
Product or service knowledge	Commercial product and service characteristics	Company representative, company literature

#### How was the information or experience encountered?

It was evident from the data that senses such as smell and touch were used by healthcare professionals as they encountered sources of knowledge, in addition to seeing and hearing. Additionally, there were clearly internal, i.e. non-sensory means of encountering knowledge such as reflecting on one’s experience (Table 3).

**Table 3:** A classification of knowledge encounters: how the knowledge is encountered (facets, descriptions and examples)

How encountered	Description	Examples from included studies
Verbal communication	Through voice either face-to-face or via a medium, such as a telephone	Informal conversation with colleagues, telephone hotline to specialist
Written format	Through print either in paper or electronic form	Company literature, textbooks
Visual	Through observation either directly or through some form of print or electronic medium	Video
Haptic	Through touch	The patient’s body
Olfactory	Through smell	Reading the patient using senses (smell, listening, watching, touch)
Internal reflection	Through conscious exploration of personal thoughts and experiences	Nurses’ known explorations

#### What was the mode of the encounter?

It was evident that some encounters took place in person and some remotely or through a medium. Where the encounter was not in person, electronic means were dominant ways of bridging the gap.

Technology would be used in many settings to augment the sharing of information, for example at a conference, but the distinction here has more to do with whether the individual is present in person rather than using a medium to communicate (Table 4).

**Table 4:** A classification of knowledge encounters: mode of information or experiential knowledge encounter (facets, descriptions and examples)

Mode of encounter	Description	Examples from included studies
Using electronic technology	Through any form of electronic medium	Email contact with specialist, telephone hotline to specialist, medical websites
Not using electronic technology	Through non-electronic media or by being present in person	Conference, local audit, hand over, handbooks

#### From whom did the information originate or with whom was the experience?

Our thematic organisation of the people from whom information originated or with whom there was an experience resulted in eight facets (Table 5). Whilst in this table the facets are individual, we conceive of the terms being used in the plural too, e.g. “salesperson” as the facet for the source “pharmaceutical/product literature”.

**Table 5:** A classification of knowledge encounters: from whom did the knowledge originate (facets, descriptions and examples)

From whom	Description	Examples from included studies
Practitioner	A healthcare practitioner from any field	Colleague, colleagues via internet, clinical leaders, personal clinical experience, journal club, email discussion list, professional association
Non-practitioner (colleague)	A colleague who is not a healthcare practitioner and who may work within or without the same organisation	Social services, non-medical personnel
Patient	A person or group of patients receiving healthcare interventions	Patients, patient questioning, patients’ experience of illness
Researcher	A person or group of people who have investigated something in an organised and systematic way	Thesis, journal
Educator	A person or organisation providing instruction	Teacher, educational booklet, laboratory manual, patient information
Regulator	A person or organisation that directs or regulates some aspect of healthcare practice	Government documents, health policy
Employer	An individual or organisation that employs the healthcare professional	Policy and procedure manuals, local guidelines
Salesperson	An individual or organisation that sells good or services	Company literature, company representative, drug company functions

#### How many participants were there?

In addition to determining the role of the individual or organisation involved in sharing the information, it was evident that some of the encounters with sources might involve an individual alone, with one or two others, or as part of a larger group. Social learning is believed to be an important element of how individuals acquire new knowledge, particularly in practice [125–127], and therefore, we felt that this facet of a knowledge encounter was an important one to capture (Table 6).

**Table 6:** A classification of knowledge encounters: how many people were involved (facets, descriptions and examples)

How many	Description	Examples from included studies
Alone	An individual encountering information or experiential knowledge without others present either physically or virtually	Personal development of acquired knowledge through experience and prior knowledge
A couple	Two people involved in the knowledge encounter	Informal conversation with colleagues
A group	Three or more people involved in the knowledge encounter	Email discussion lists, seminar

#### Where did the encounter take place?

For this category, we tried to distil from the sources identified a sense of place in which the knowledge encounter could occur. The setting was in part determined by the intention, e.g. whether a meeting was intended to be an educational event or if it was intended to be social but where new information was encountered nonetheless (Table 7).

**Table 7:** A classification of knowledge encounters: where did the knowledge encounter take place (facets, descriptions and examples)

Where	Description	Examples from included studies
Work	Setting in which the healthcare professional works	Grand rounds
Educational	Setting intended for educational activity rather than work	Conferences
Domestic	Home	Online continuing education, chatrooms (if carried out at home)
Social	A setting given over to social activities rather than educational or work	Professional societies (when primary reason for meeting is social)

We have summarised the categories and facets in Fig. 2.

## Discussion

We have conducted a systematic mixed studies review to identify all encounters with information and experiences reported in the healthcare literature and identified 80 qualitative, quantitative and mixed methods studies that reported on such encounters. We have then developed a faceted classification to describe what we term *knowledge encounters* using a mixed didactic-inductive process. The classification has six categories that describe different aspects of any encounter: what information or experience was encountered, how the information or experience was encountered, what the mode of encounter was, from whom did the information originate or with whom was the experience, how many participants there were, and where the encounter took place. Each category is described by key facets that enable a deeper understanding of the actual knowledge encounter.

### Discussion of the generation of facets

Higgs and Titchen [128] describe three knowledge types in professional practice: theoretical or scientific knowledge, craft knowledge and personal knowledge. Similarly, Kemmis proposes that there is public knowledge (theoretical, scientific), action (as an object of knowing or thought) and practice (theory) [127]. Eraut divides knowledge into codified (also known as public or propositional knowledge) and personal [129]. Gabbay and Le May identified six knowledge types from their work with healthcare providers over many years. These knowledge types were experiential, research, theoretical, policy, custom and practice and trial and error [18, p 109]. We drew on all of these in developing the classification, but in practice, we found at times that we were unable to determine whether, or how much, knowledge encounters were with each facet we described. For example, *Guidelines* are known to incorporate both research and the developers’ expertise. Further, a reader of a *textbook* might be unaware what was based on empirical research, what on theory and what on the personal experiences of the authors. Whilst it might be evident that there are these distinctions in some cases (e.g. when reading a research article in a journal or an *evidence summary*), we adopted the common denominator of “codified knowledge”.

One source of information that arose from the data was that produced by commercial companies, often for the purpose of marketing. It is not clear whether this has been subjected to quality control in the sense of codified knowledge. We therefore included an additional category of “product or service knowledge” though we acknowledge that there may be occasions when the product knowledge truly reflects codified knowledge, i.e. when it is based on peer-reviewed research.

There were multiple roles individuals or organisations could be envisaged to perform depending on the context of the encounter. From a relational perspective, a “colleague*”* might be someone whose “practice is observed*”* or with whom an “informal conversation*”* is had. Colleagues might take on the role of an educator or as a “mentor*”* or “peer opinion leader*”*. They might also be an employer and responsible for creating “locally developed guidelines*”*. In many cases, it was not possible for us to define the role due to lack of contextual information, but there were sufficient examples of different roles across the included studies for us to develop a number of facets to describe the various roles others play in knowledge encounters.

Given that an individual can perform several different roles, we defined this category according to the primary role taken by the individual(s) or organisation at the moment of information or knowledge encounter. Thus, a “journal club*”* may be dependent on the interpretations of fellow practitioners, whilst the reading of a research article in a “journal*”* alone would entail the source being a researcher.

There was a challenge in how to classify non-human sources such as journal articles and “video”. We determined that the primary role of the individual(s) or organisations creating the particular information source would follow the same categories as for the in-person encounters. Thus, a research article may be written by a researcher who might write another article that explains a clinical technique as an educator. The descriptive facets for this category can be seen in Table 5.

These facets could equally be ascribed to the self or to other colleagues. In such a case, personal reflection by a healthcare professional on their practice would be described by the facet “*practitioner”*.

### Preliminary testing of the classification with dentists

The faceted classification developed here is a complex one. We want to test its feasibility in future studies. We created a series of five scenarios based on a composite of the sources extracted for this review, for dentists to work through, with a description of what was intended by the different facets [130]. Each scenario used a mixture of photos and text of a dentist encountering information or experiential knowledge. After brief training on the use of the classification, four dentists working in general dental practice independently classified the 5 scenarios according to the type of knowledge encounter. There was good agreement (75–100%) for all but the “setting” category in two of the scenarios, when there was 50% agreement. The dentists fed back that this tool was straightforward to use once they had undergone the training.

### Limitations

There are a number of potential limitations associated with this review.

The search for studies used a combination of formal search strategy and citation searching. It is possible that we have missed potentially relevant studies because authors have used terms we did not include in the search strategy and because we confined the eligible studies to English. However, as we were not seeking to quantify the encounters with knowledge sources, we feel that we reached a saturation of both classifications of knowledge encounters and descriptions of their key facets in the studies we did identify.

The screening of titles and abstracts was conducted by a single reviewer. This increases the possibility that potentially eligible studies were not included. Similarly, it is a limitation that the coding and design of the classification were done by one author.

The discreet facets identified may not reflect all the facets that a particular knowledge encounter may include. Thus, users of the classification will have to make a choice about which facet is most appropriate of all those that could be used.

We have also identified limitations of the studies included. The literature on knowledge sources identified here has been largely dependent on recall of exposure to different sources. Apart from three studies that used brief periods (4 h or half a day) of observation [53, 77, 101], all of the quantitative data has depended on recall. This review was not concerned with quantifying the proportion of encounters ascribed to each source, but due to the impact of recall bias [131], we may not have a complete picture of sources of information encountered by healthcare professionals. In the qualitative studies, much more contextual information was given to understand what the encounter with a particular source meant to the clinician. Finally, several of the closed question surveys we identified appeared not to follow a particular theoretical structure, and we were unable to identify one that had all of the knowledge source encounters identified in this review.

We used the MMAT to assess the quality of the studies. We value the attempt by the authors of MMAT to provide a means for evaluating and comparing the quality of different study designs when conducting mixed studies reviews. However, whilst it was apparently straightforward to use with individual studies, we found that we were unsure what the results meant when compared, in particular, to studies conducted within different research paradigms where “quality” can mean something very different [132].

### Implications for implementation science

There is a growing body of evidence to support the assertion that healthcare professionals’ knowledge-in-practice—the knowledge that is used as they practice—is influenced by interactions with a variety of formal and informal sources of information and experience. However, as shown in this review, there has been relatively little real-time empirical study of the ways in which this happens. We think that by studying the ways in which healthcare professionals’ knowledge-in-practice is influenced by what they encounter and how they encounter it, we can begin to better understand how to work with the grain of practice to develop systematic approaches to contribute to achieving implementation science’s aspiration of promoting the uptake of research and other evidence-based findings into practice [16].

From their ethnographic work in general medical practices, Gabbay and Le May coined the term mindlines to describe the internal, personal, tacit guidance medical practitioners used as they went about their practice. Mindlines develop as healthcare professionals interact with, and make sense of, multiple sources of information and experiences. Recognising that quantitative methods can rarely develop the rich understanding of individual practices that ethnographic methods can, we nonetheless think that the classification described here will allow for longitudinal survey tools to be developed that capture rich contextual data of how healthcare professionals develop their knowledge-in-practice in a variety of contexts.

With the increasing availability and functionality of digital means to record experiences in real time [133], there is an opportunity to study larger numbers of healthcare professionals in any number of settings as they go about their lives. Observational methods are limited not only by the large amount of time needed and ability for a researcher to be in only one place at a time but also by restrictions of access to, for example, peoples’ homes and social gatherings where knowledge encounters are bound to occur. Real-time data capture has been used to study a wide range of health and social topics [134–137], and we envisage research that explores knowledge encounters in a similar way. This could be done qualitatively, with participants recording in their own words their experience of the encounter. However, by using a classification to describe the knowledge encounters, participants may be able to record encounters more quickly and record key characteristics of the encounter. Meanwhile, researchers would be able to gather larger quantities of data and analyse it much more quickly. Through a better understanding of what, how, where and with whom information and experiences diffuse into practice, it is hoped that we can develop new implementation approaches to promote research uptake alongside other information and experience as healthcare professionals develop their knowledge-in-practice.

Further, development of this longitudinal approach could explore whether, and how, particular facets of knowledge encounters are associated with changes in knowledge-in-practice and how these facets change, for example as healthcare professionals mature. Implementation researchers might then develop interventions that leverage off these facets to help improve uptake of evidence-based practices. For example, if knowledge encounters that share the facets of individualised codified knowledge, verbal, non-electronic, with practitioners, in social settings and occur in groups are associated with more change in knowledge-in-practice, can we adopt, adapt or design new opportunities for professionals to experience knowledge encounters with research based on these, rather than on facets not associated with changes in knowledge-in-practice?

Finally, whilst classifications function as descriptive and explanatory frameworks for ideas or phenomena, they also act like theories do in providing the basis for the generation and testing of new ideas [138]. We think that by describing what is a complex phenomenon using a faceted classification, implementation researchers may find new ways of thinking about knowledge-in-practice and how to work effectively with it to promote the use of research and other evidence-based findings in practice.

## Conclusions

Healthcare professionals encounter information and experiences that may change their knowledge of patient care in many different ways. We have developed a classification to organise and describe the complexity of how healthcare professionals encounter information and experiences. We have identified six key classifying questions to understand the context in which information and experiences are encountered and described important facets for each of these. This novel faceted classification for knowledge encounters has been developed as a tool for future observational studies of healthcare professionals. Future research should explore how this classification of knowledge encounters can deepen our understanding of how healthcare professionals learn and develop their knowledge-in-practice. Over time, we want to be able to facilitate knowledge encounters that improve the uptake of evidence-based practices in healthcare.

## Additional files

Additional file 1: Search strategy. (PDF 448 kb)
Additional file 2: Included studies. (PDF 598 kb)
Additional file 3: Information and experiences extracted from included studies. (PDF 329 kb)

## Abbreviations

BC2: Bliss Bibliographic Classification
MMAT: Mixed Methods Appraisal Tool
