# Supplementary material for: Describing knowledge encounters in healthcare: a mixed studies systematic review and development of a classification
Source: Implement Sci. 2017 Mar 14;12:35. doi: 10.1186/s13012-017-0564-1 (PMC5351057; doi:10.1186/s13012-017-0564-1)
Supplement: Additional file 1: — Search strategy. (PDF 448 kb) [file 13012_2017_564_MOESM1_ESM.pdf]

Additional file 1 – Search strategy

OVID Medline and OVID Embase were searched together using the following strategy:

#	Searches	Results
1	(seek or seeking).mp.	162033
2	(search or searching).mp.	402232
3	(utilization or utilisation).mp.	403017
4	exchange.mp.	476370
5	(share or sharing).mp.	228699
6	resource$.mp.	441850
7	source$.mp.	959411
8	doctor$.mp.	274357
9	(nursing or nurse$).mp.	1169812
10	(dental or dentist$).mp.	738455
11	(veterinary or vet or vets).mp.	115311
12	(podiatry or podiatrist$).mp.	5685
13	(therapy or therapist$).mp.	5024767
14	(physiotherapy or physiotherapist$).mp.	80958
15	psychologist$.mp.	26774
16	surgeon$.mp.	346420
17	evidence.mp.	2449208
18	knowledge.mp.	902214
19	information.mp.	1938096
20	17 or 18 or 19	4886998
21	1 or 2 or 3 or 4 or 5 or 6 or 7	2840999

22	8 or 9 or 10 or 11 or 12 or 13 or 14 or 15 or 16	7427020
23	((evidence or knowledge or information) adj1 (seek or seeking or (search or searching) or (utilization or utilisation) or exchange or (share or sharing) or resource$ or source$)).mp.	32705
24	22 and 23	8168
25	limit 24 to english language	7579
26	limit 25 to human	6553

Note: mp=ti, ab, sh, hw, tn, ot, dm, mf, dv, kw, nm, kf, px, rx, ui

Subject headings (sh above) are included in the search along with keywords.
