# Supplementary material for: Describing knowledge encounters in healthcare: a mixed studies systematic review and development of a classification
Source: Implement Sci. 2017 Mar 14;12:35. doi: 10.1186/s13012-017-0564-1 (PMC5351057; doi:10.1186/s13012-017-0564-1)
Supplement: Additional file 2: — Included studies. (PDF 598 kb) [file 13012_2017_564_MOESM2_ESM.pdf]

Additional file 2 – Included studies

Author	Year	Study method	Tool type	Recall or real-time	Tool creator / name	Qual/ Quant/ Mixed	Cross-sectional/ longitudinal	Country	Setting	Healthcare grp	n=	MMAS quality rating (max ****)
**Al-Ghabeesh**	2012	Survey	Questionnaire	Recall	Estabrooks'	Quantitative	Cross	Jordan	Hospital	Nurses	555	**
**Amary-Risch**	2009	Survey	Web-based questionnaire	Recall	Docstyles survey	Quantitative	Cross	USA	Primary care + hospital	Doctors	1500	*
**Andrews**	2005	Survey	Mailed questionnaire	Recall	Author designed	Quantitative	Cross	USA	Primary care	physicians, nurse practitioners, and physician assistants	59	*
**Ankem**	2003	Interviews	Telephone interview	Recall	Author designed	Mixed	Cross	USA	Hospital	Interventional radiologists	32	**
**Apalayine**	1996	Survey	Self-administered questionnaire	Recall	Unclear	Quantitative	Cross	Ghana	Primary care	Nurses and midwives	47	**
**Arroll**	2002	Observation + survey	Observation and questionnaire	Both	None for sources	Quantitative	Both	New Zealand	Primary care	Doctors	50	***

**Asefzadeh**	2008	Survey	Questionnaire	Recall	Unclear	Quantitative	Cross	Iran	All	Doctors, dentists	339	*
**Barley**	2008	Interviews	Semi-structured interviews with topic guide	Recall	N/A	Qualitative	Cross	England	Hospital or primary care - unclear	Psychiatrists	22	***
**Bennett**	2005	Survey	Faxed Questionnaire	Recall	Author designed	Quantitative	Cross	USA	Primary care	Doctors	457	**
**Bennett**	2006	Survey	Faxed Questionnaire	Recall	Author designed	Quantitative	Cross	USA	All	Doctors	2500	***
**Bernard**	2012	Survey	Online questionnaire	Recall	Author designed	Quantitative	Cross	France	Primary care	Doctors	721	*
**Beualieu**	2008	Case study	Interviews	Recall	N/A	Qualitative	Cross	Canada	Primary care	Doctors	22	****
**Bonner**	2011	Interviews	One to one interviews	Recall	N/A	Qualitative	Cross	Australia	Hospital	Renal nurses	6	**
**Botello-Harbaum**	2013	Survey	Online or printed questionnaire	Recall	Author designed	Quantitative	Cross	USA	Primary care	Dentists	950	**
**Bringsvor**	2014	Interviews	Focus groups	Recall	N/A	Qualitative	Cross	Norway	Hospital	Intensive care nurses	20	****

**Bryant**	2004	Interviews	One to one and group interviews	Recall	N/A	Qualitative	Cross	UK	Primary care	Doctors	20	**
**Buckley**	2014	Survey	Online questionnaire	Recall	Author designed	Quantitative	Cross	Australia	Not specified	Nurse practitioners	208	**
**Butzlaff**	2002	Survey	Questionnaire	Recall	Author designed	Quantitative	Cross	Germany	Primary care	Doctors	72	****
**Callen**	2008	Survey	Questionnaire	Recall	Author designed	Quantitative	Cross	Mongolia	Hospital	Doctors	229	**
**Chang**	2009	Survey	Emailed questionnaire	Recall	Author designed	Quantitative	Cross	South Korea	Not specified	Dentists	840	*
**Chew-Graham**	2008	Interviews	Semi-structured interviews	Recall	N/A	Qualitative	Cross	UK	Primary care	Doctors	14	***
**Clarke**	2002	Case study	Interviews, focus group	Recall	N/A	Qualitative	Cross	UK	Primary care, hospital	Nurses	41	**
**Codgill**	2003	Survey and interviews	Mailed questionnaire and individual interviews	Recall	Author designed	Mixed	Cross	USA	Primary care	Nurse practitioners	14	**
**Curley**	1990	Survey	Questionnaires	Recall	Unclear	Quantitative	Cross	USA	Hospital, primary care	Doctors	228	**
**Dee**	1993	Case study	Interviews	Recall	N/A	Qualitative	Cross	USA	Hospital, primary care	Doctors	12	**

**Elliott**	1991	Survey	Questionnaire	Recall	Author designed	Quantitative	Cross	USA	Not specified	Doctors	150	**
**Estabrooks**	1998	Survey	Questionnaire	Recall	Author designed	Quantitative	Cross	Canada	Any	Nurses	600	***
**Estabrooks**	2005 b	Survey	Questionnaire	Recall	Author designed	Quantitative	Cross	Canada	Hospitals	Nurses	230	**
**Estabrooks**	2005 a	Ethnographic	Observation; interview; card sort	Both	N/A	Qualitative	Both	Canada	Hospitals	Nurses	7 units; no info on participants	**
**Fairhurst**	1998	Case study	Interviews	Recall	N/A	Qualitative	Cross	UK	Primary care	Doctors	24	***
**Gabbay**	2004	Ethnographic	Interviews, observation, documentary review	Both	N/A	Qualitative	Longitudinal	UK	Primary care	Doctors,nurses, phlebotomist	13	****
**Gagliardi**	2008	Survey	Mailed questionnaire	Recall	Author designed	Quantitative	Cross	Canada	Hospital, community	Doctors	170	**
**Gavino**	2013	Survey	Internet and paper questionnaire	Recall	Modified Olson, R., James, E. & Fontelo, P. [1]	Quantitative	Cross	Philipines	Not specified	Doctors	156	*

[1] Information Access Trends Among Clinicians at a Community Hospital in Northeast Georgia. AMIA 2010 Symposium Proceedings [Internet]. 2010 [cited 2011 August 2]. pp. 1198. Accessible at: http://proceedings.amia.org/120up3/1?highlightText=olson%20fontelo&

**Gerrish**	2008	Survey	Questionnaire	Recall	Developing Evidence-Based Practice (DEBP) Questionnaire - Sources section based on Estabrooks'	Quantitative	Cross	UK	Hospital	Nurses	598	**
**Gonzalez-Gonzalez**	2007	Observation and interview	Video and interview	Both	N/A	Quantitative	Longitudinal	Spain	Primary care	Doctors	112	**
**Gravois**	1995	Survey	Questionnaire	Recall	Author designed	Quantitative	Cross	USA	Various	Hygienists	44	**
**Jackson**	2007	Survey	Postal Questionnaire	Recall	Author designed	Quantitative	Cross	UK	Primary care + hospital	Doctors, nurses	54	**
**James**	2010	Ethnography	Observation, interviews, conversation	Both	N/A	Qualitative	Longitudinal	Sweden	Hospital	Nurses	25	****
**Kosteniuk**	2006	Survey	Questionnaire	Recall	Author designed	Quantitative	Cross	Canada	Various	Nurses	3933	***
**Kosteniuk**	2013	Survey	Questionnaire	Recall	Author designed	Quantitative	Cross	Canada	Primary care	Doctors	331	***
**Martinez-Silveira**	2008	Survey	Questionnaire	Recall	Author designed	Quantitative	Cross	Brazil	Hospital	Doctors	85	*
**McCord**	2007	Survey	Observation	Recall	N/A	Quantitative	Cross	USA	Hospital	Doctors	25	**

**McGettigan**	2001	Survey	Questionnaire	Recall	Unclear	Quantitative	Cross	Australia	Hospital and primary care	Doctors	226	**
**McKnight**	2006	Ethnography	Observation, interviews	Both	N/A	Qualitative	Longitudinal	USA	Hospital	Nurses	6	***
**Mills**	2009	Survey	Questionnaire	Recall	Developing Evidence-based Practice Questionnaire (DEPQ) (Gerrish et al. 2007)	Quantitative	Cross	Australia	Primary care	Nurses	590	***
**Nail-Chiwetalu**	2007	Survey	Questionnaire	Recall	Author designed based on Powell CA, Case-Smith J. [2]	Quantitative	Cross	USA	Various	Speech and language pathologists	208	**
**Neher**	2014	Interviews	One to one	Recall	N/A	Qualitative	Cross	Sweden	Hospital and primary care	Rheumatology nurses	12	***
**Nichols**	2008	Ethnography	Observation and interview	Recall	N/A	Qualitative	Cross	UK	Hospital	Doctors, nurses	14	****
**Nieri**	2008	Survey	Questionnaire via telephone interview	Recall	Unclear but suspect author designed	Quantitative	Cross	Italy	Primary care	Dentists	123	***

[2] Information literacy skills of occupational therapy graduates: a survey of learning outcomes. J Med Libr Assoc 2003 Oct;91(4):468–77.

**Norbert**	2013	Survey	Questionnaire	Recall	Unclear but suspect author designed	Quantitative	Cross	Tanzania	Hospital	Doctors	215	*
**Northup**	1983	Critical incident technique	Telephone interview	Recall	Author designed	Quantitative	Cross	USA	Varios	Doctors	293	***
**Nylenna**	2000	Survey	Postal Questionnaire	Recall	Unclear	Quantitative	Cross	Norway	Primary car and hospital	Doctors	1276	**
**O'Leary**	2012	Interviews, Survey	Interviews, questionnaire	Recall	Author designed	Mixed	Cross	Ireland	Various	Nurses	29 intervie w 377 survey	***
**Oliveri**	2004	Survey	Questionnaire	Recall	Unclear but suspect author designed	Quantitative	Cross	Denmark	Hospital	Doctors	226	*
**Ozsoy**	2008	Survey	Questionnaire	Recall	Estabrooks'	Quantitative	Cross	Turkey	Hospital	Nurses	498	**
**Papp**	2002	Survey	Questionnaire	Recall	Author designed	Quantitative	Cross	USA	Primary care	Doctors	105	*
**Peay**	1984	Survey	Interviews	Recall	N/A	Quantitative	Cross	Australia	Primary care	Doctors	124	*
**Pelzer**	1991	Survey	Questionnaire	Recall	Author designed	Quantitative	Cross	USA	Primary care	Vets	287	**
**Perzeski**	2012	Survey	Questionnaire (web)	Recall	Author designed	Quantitative	Cross	USA	Various	Podiatrists	143	*

**Ramos**	2003	Observation	Observation	Real-time	N/A	Quantitative	Longitudinal	USA	Primary care	Doctors	38	*
**Rappolt**	2002	Ethnography	Interviews	Recall	N/A	Qualitative	Cross	Canada	Primary care + hospital	Rehabilitation therapists	24	***
**Robertson**	2011	Ethnography	Interviews	Recall	N/A	Qualitative	Cross	Australia	Primary care	Doctors	40	***
**Sarbaz**	2016	Survey	Questionnaire	Recall	Author designed	Quantitative	Cross	Iran	Hospital	Nurses	131	*
**Secco**	2006	Survey	Questionnaire	Recall	Nursing Information Use Survey (NIUS) - Winnipeg Childrens' Hospital	Quantitative	Cross	USA	Hospital	Nurses	113	*
**Selvi**	2002	Survey	Questionnaire	Recall	Author designed	Quantitative	Cross	Turkey	Primary care	Dentists	133	**
**Shelstad**	1996	Survey	Questionnaire	Recall	Unclear but suspect author designed	Quantitative	Cross	USA	Primary care + hospital	Doctors	99	**
**Sibbald**	2013	Questionnaire, Interviews	Questionnaire, Interviews	Recall	Social Network Analysis Questionnaire - Author designed	Qualitative	Cross	Canada	Primary care	Nurses, doctors, allied healthcare	28	**

**Stetler**	1991	Interviews	Semi-structured interviews	Recall	N/A	Quantitative	Cross	USA	Hospital	Nurses	24	*
**Straub-Morarend**	2011	Survey	Questionnaire	Recall	Author designed	Quantitative	Cross	USA	Primary care	Dentists	518	*
**Strother**	1986	Survey	Questionnaire	Recall	Author designed	Quantitative	Cross	USA	Primary care + hospital	Dentists	344	**
**Tabatabaei-Malazy**	2912	Survey	Questionnaire	Recall	Author designed	Quantitative	Cross	Iran	Any	Doctors	319	**
**Thompson**	2001a 2001b	Case study	Observation, interviews, document analysis, Q analysis	Both	N/A	Qualitative	Both	UK	Hospital	Nurses	108	***
**Timpka**	1989	Survey	Questionnaire	Recall	Author designed	Quantitative	Cross	Sweden	Primary care	Doctors	186	**
**Urquhart**	1994	Vignettes	Vignettes	Recall	N/A	Qualitative	Cross	UK	Hospital and primary care	Nurses	64	***
**Vollmar**	2009	Survey	Questionnaire	Recall	Unclear but suspect author designed	Quantitative	Cross	Germany	Primary care	Doctors	264	**

**Wardh**	2009	Survey, interviews	Postal Questionnaire Focus group Interviews	Recall	Unclear but suggests author designed~~Unclear but suspect author designed~~	Mixed	Cross	Sweden	Primary care; hospital	Dentists	Questionnaire: 177. In focus groups: 15	**
**Warren-Findlow**	2010	Focus groups, interviews	Focus groups, interviews	Recall	N/A	Qualitative	Cross	USA	Primary care	Doctors, nurses, doctors' assistants	49	**
**Weng**	2013	Survey	Questionnaire	Recall	Author designed	Quantitative	Cross	Taiwan	Hospital	Doctors, nurses, pharmacists, physical therapists, technicians	6160	***
**Yadav**	2012	Survey	Development of Evidence-Based Practice Questionnaire (Gerrish)	Recall	Developing Evidence-based Practice Questionnaire (DEPQ) (Gerrish et al. 2007)	Quantitative	Cross	Ireland	hospital, primary care	Nurses	145	**
**Yousefi-Nooraie**	2007	Survey	Questionnaire	Recall	Unclear	Quantitative	Cross	Iran	hospital	Doctors	250	*
