# Supplementary material for: Describing knowledge encounters in healthcare: a mixed studies systematic review and development of a classification
Source: Implement Sci. 2017 Mar 14;12:35. doi: 10.1186/s13012-017-0564-1 (PMC5351057; doi:10.1186/s13012-017-0564-1)
Supplement: Additional file 3: — Information and experiences extracted from included studies. (PDF 329 kb) [file 13012_2017_564_MOESM3_ESM.pdf]

Additional File 3 - Information and experiences extracted from included studies

Peers / colleagues
Observation of others' practice
Formalised supervision
Systematic self-evaluation
Specialist (including consultants in hospitals)
Email contact with specialist
Telephone hotline to specialist
University colleagues
Informal conversation with colleagues
Personal contacts
Colleagues via internet
Mentor / senior clinician
Peer opinion leaders
Specialist dentist
"Dental practice expert"
CNS/CNE
Preceptor/resource persons
Non-medical personnel
Unit managers
Practice development team
Students
Teachers
Preceptor / supervising doctor
Clinical leaders
Physicians (discussions with)
Residents
Grand rounds
Referral information & feedback from colleagues
Informal networks
Specialty groups
Allied health workers
"Other" healthcare professionals
Prescribing advisors
Other organisations
Social services
Local care delivery (arrangements)
Local service evaluations

Local prescribing culture
Pharmaceutical companies
Pharmaceutical companies' websites
Pharmaceutical / product literature
Drug company functions
Company reps
Company literature
Advertising (aimed at healthcare professionals)
Direct mail
Manufacturer
Drug information
Package insert
Medication / treatment pamphlets
Drug reference sources - print format
Drug reference sources - e-format
Filtering services
Clinical calculators
Computer decision aid
Patients
Patient questioning
Patient record
Patients' experiences of illness
Observing patients in public space
Patient surveys
Patients’ families
Health information service for patients
The body - own
The body - other nurses'
The body - patient's
Reading the patient using senses (smell, listening, watching, touch)
Conferences / conventions / congresses
Seminars
Workshops
Courses - lecture-based
Courses - hands-on
Lectures sponsored by workplace
Symposiums
Continuing education - local
Continuing education - national
Continuing education - non-specific
CME articles
Departmental courses
Inservice education at work
Professional meetings including association meetings (national and international)

In-services
Internships
Orientations
Rounds
Meetings
Journal clubs
Quality circles
Study club
What has worked / not worked before
Personal development of acquired knowledge through experience and prior knowledge
Side-effects in patients
Personal clinical experience
Personal experience of patients' experiences of illness
Nurses’ known explorations
Intuition
Setting knowledge arising from social & clinical pressure, expectations and events
Patients’ charts
Policy & procedure manuals
National health policy
Government documents
Video & other media including audio tapes
Thesis or dissertation
Newsletters
Communication books
Bulletin boards
Electronic resources (including CD ROM)
Internet
Computer information networks
Intranet
Medical websites (incl. WebMD)
Organisational websites
Patient group websites e.g. dementia
Internet - public sites
Internet - databases
Internet - search engines
Computer programme
Computer databases
Personal digital assistant
Medline / index medicus (offline)
Point of care databases
Chatrooms
Email discussion lists
Email use
Online continuing education

CD Medical textbook
Patient information
Patient-specific information via internet
prescription / patient orders via internet
Books, journals, previous research
Textbooks (not grouped with other publications) including 'personal library'
Textbooks - electronic
Pocket reference / book
Handbooks or manuals
Technical reports
Diagnostic laboratory
Laboratory manual
Educational booklet or brochure
Informal documents
Journals (not grouped with other publications) including online journals
Journals - editorials
Summaries of studies including structured abstracts
evidence-based clinical decision support resource
Reviews
Case studies
Professional association updates via internet
Professional association
Professional association website
Unions
Professional newsletters
Brief updates
Professional organisations / societies
External (professional) bodies
Clinical guidelines
Locally developed guidelines or protocols
Evidence-based practice
Professional literature
Non-peer reviewed dental information
Free professional magazines
Public media
Personal beliefs
Personal non-clinical experience
Basic education (at nursing / medical / dental / vet etc. school)
Common sense
Training
Own notes
Local audit
Practice or intradepartmental meetings
Group/local norms

The ways it has always been done
Handover
Chiefs / local heads
Local community
Library / Information centre
Librarian
Electronic documents (offline e.g. on disk)
Hospice
Organisational features
Wall chart
